# Inhibition of the RIP3/MLKL/TRPM7 necroptotic pathway ameliorates diabetes mellitus-induced erectile dysfunction by reducing cell death, fibrosis, and inflammation

**DOI:** 10.3389/fphar.2024.1436013

**Published:** 2024-09-12

**Authors:** Lipan Niu, Pei Yang, Bingbing Zhu, Xiufang Jin, Chengxia Yang, Xijia Zhang, Yulian Liu, Rui Zhang, Fengxia Liu

**Affiliations:** ^1^ Department of Human Anatomy, College of Basic Medicine Science, Xinjiang Medical University, Urumqi, China; ^2^ Xinjiang Key Laboratory of Molecular Biology of Endemic Diseases, Urumqi, China

**Keywords:** necroptosis, RIP3/MLKL/TRPM7, corpus cavernosum smooth muscle cells, diabetes mellitus-induced erectile dysfunction, RIP3 inhibitor, Yimusake

## Abstract

Diabetes mellitus-induced erectile dysfunction (DMED) is a common complication in patients with diabetes mellitus. Necroptosis is regarded as a form of cell death that is intimately associated with the inflammatory response, which is not only initiated by inflammatory factors such as TNF-α, but also triggers the inflammatory cascade through the rupture of the dying cell. There is no definitive study on the role of necroptosis in the pathological process of DMED. In light of the pathological features of high inflammation levels in DMED patients, we assessed whether the necroptosis plays an important role in the course of DMED. Our study revealed that penile tissues of DMED rats showed high levels of key necroptosis factors such as receptor-interacting protein kinase 3 (RIP3), mixed-lineage kinase domain-like protein (MLKL), and transient receptor potential melatonin 7 (TRPM7). Furthermore, the inhibition of necroptosis with a receptor-interacting protein kinase 3 (RIP3) inhibitor or Yimusake (a common herbal remedy for ED) effectively rescued damage to corpus cavernosum smooth muscle cells (CCSMC) under high glucose conditions. Our findings suggest that inhibition of the RIP3/MLKL/TRPM7 necroptotic pathway could effectively ameliorate CCSMCs fibrosis and death induced by high glucose and inhibited the inflammatory response.

## 1 Introduction

Erectile dysfunction (ED) is a prevalent male condition where the penis cannot attain and/or sustain an erection adequate for satisfactory sexual intercourse in response to sexual stimulation for a minimum of 6 months (Mazzilli, 2022; Yoo et al., 2016). The onset of ED can be insidious and has a gradual progression, resulting in a substantial impact on the psychological well-being, partnership, and overall quality of life for the man affected and his partner. As a frequent complication of diabetes mellitus (Yuan et al., 2020), the incidence rate of erectile dysfunction in diabetes patients is three times that of the general population (Xu et al., 2022). Patients with diabetes mellitus-induced ED (DMED) tend to exhibit more severe symptoms that are difficult to manage. Phosphodiesterase type 5 inhibitors, as the first-line treatment for ED, appear to have limited effectiveness in treating DMED patients (Cayetano-Alcaraz et al., 2023; Peng et al., 2022). This can be attributed to the lack of a complete and comprehensive understanding of the disease’s intrinsic developmental laws and internal mechanisms. Studies have shown that hyperglycaemia-induced reduction in the number and functional impairment of corpus cavernosum smooth muscle cells (CCSMCs) is a major causal factor in DMED (Li et al., 2021). However, the precise mechanism behind the impairment remains unclear.

Necroptosis, a recently discovered form of programmed cell death that exhibits both necrotic and apoptotic characteristics. Receptor-interacting protein kinase 3 (RIP3) has been identified as a vital regulatory protein in the necroptosis pathway (Morgan and Kim, 2022). Studies indicate that RIP3 recruits and phosphorylates mixed lineage kinase domain like protein (MLKL), resulting in the translocation of polymerised MLKL to either the cell membrane or nuclear membrane (Martinez-Osorio et al., 2023). This then causes Ca^2+^ efflux, culminating in cell disintegration and death. However, there is disagreement concerning the precise downstream signalling pathways of MLKL (Guo et al., 2019; Gutierrez et al., 2017). Currently, necroptosis mediated by RIP3 has been observed in various cardiovascular diseases such as atherosclerosis and cardiac hypertrophy (Xue et al., 2022). Although ED and cardiovascular disease share a common pathophysiological basis, the role of RIP3-mediated necroptosis in the regulation of DMED remains unclear.

Hence, the research investigated the effect of RIP3-mediated necroptosis on ED in rats. This was accomplished by developing a model of DMED in rats to investigate its pathogenesis and scientific implications thoroughly. The findings will provide a reference for discovering a more efficient and secure pharmacological treatment.

## 2 Materials and methods

### 2.1 Experimental animals

All rats in the study were obtained from the Experimental Animal Centre of Xinjiang Medical University. 60 six-week-old male Sprague-Dawley rats exhibiting normal sexual function, as confirmed by the apomorphine (APO) experiment (Heaton et al., 1991), all weighing (200 ± 20) g. All rats were reared with free access to water and food, and received regular 12/12 h light/dark cycle at an ambient temperature of 20°C–24°C with relative humidity of 40%∼60%. This experiment was approved by the Experimental Animal Ethics Committee of Xinjiang Medical University (No. IACUC-20210405-7).

### 2.2 Establishment and screening of the DMED rat model

Ten male rats were selected at random to form the normal control group (labeled *N*), while the remaining 50 rats were identified as the diabetic group. Following 2 weeks of acclimatisation, a diabetic model was prepared by administering two low-dose intraperitoneal injections of streptozotocin (S0130, Sigma, United States, 45 mg/kg) for 3 days. Successful diabetic models with a fasting blood glucose level greater than 16.7 mmol/L were referred after 72 h. All rats were supplied with water and food *ad libitum* for 8 weeks. DMED rats were screened together with APO experiment and paired experiment, and 24 were randomly selected and divided into four groups (n = 6 per group): DMED group, GSK872 group (a type of RIP3 inhibitor, HY101872, 1 mg/kg; labeled *RIP3i*) (Xuan et al., 2021), Yimusake group (in tablet form, Z65020144, Xinjiang, China, 250 mg/kg; labeled *Y*), GSK872 + Yimusake group (labeled *RIP3i + Y*). The intervention lasted 2 weeks. The serum glycated haemoglobin of rats was analysed by enzyme-linked immunosorbent assay kit (BC5610, Beijing Solaibao Technology Co., LTD, China). Erectile function was evaluated following intervention by recording maximum intracavernosal pressure (Max ICP) and mean arterial pressure (MAP) through cavernous nerve electrical stimulation (15 Hz; 7.5 V; 1 min), as described in reference (Zhu et al., 2023). The stimulation was repeated three times per rat (at the same voltage), with a 15-minute interval between each electrical stimulation, and ICP/MAP was calculated. Finally, rats were killed by intraperitoneal injection of pentobarbital sodium and blood from the abdominal aorta and penile tissue were collected, and stored at 4°C, −80°C and in 4% paraformaldehyde, respectively.

### 2.3 Histological alteration

#### 2.3.1 Hematoxylin-eosin (H&E) staining

The penile tissue was fixed in 4% paraformaldehyde solution for 48 h. Thereafter, the samples were routinely paraffin-sectioned, dewaxed, hydrated, and stained with hematoxylin for a duration of 2 min. They were then rinsed with distilled water and subsequently stained with eosin solution for 10 s. Finally, after the steps of dehydrating, clearing, and sealing the slices, and the samples were observed under a microscope with images collected.

#### 2.3.2 Masson trichrome staining

Masson trichrome staining was also performed using paraffin sections to reflect the level of fibrosis in penile tissue. Samples were fixed in Bouin fixative overnight after dewaxed and hydrated, and then rinsed and operated according to the instruction manual that accompanied the staining kit (G1340, Beijing Solaibao Technology Co., LTD, China). The area of penile cavernous smooth muscle (red) and collagen (blue) was measured using *ImageJ* software.

#### 2.3.3 Immunohistochemical analysis

The penile paraffin sections underwent routine dewaxing and rehydration procedures. The tissue sections were immersed in the antigen repair solution at 95°C for 30 min and allowed to cool naturally. Thereafter, the sections were incubated with primary antibodies, eNOS (1:200, AF0096, Affinity, Cincinnati, OH, United States), ET-1 (1:100, DF6125, Affinity), TNF-α (1:200, AF7014, Affinity), IL-6 (1:400, DF6087, Affinity), α-SMA (1:500, 67735-1-Ig, Proteintec), and Collagen I (1:200, AF7001, Affinity). The positive area was identified through the utilisation of image analysis software, namely, ImageJ. The area and intensity of the positive region reflected the distribution and expression level of the target protein.

#### 2.3.4 Propidium iodide staining

Propidium iodide staining was performed using paraffin sections for the detection of cell membrane breakdown in peniles. Sections were stained by adding propidium iodide stain for 15 min after dewaxed and hydrated, and then blocked using isopropanol. Tissue sections were observed under a microscope and images were captured. Counting the number of cells with ruptured cell membranes using ImageJ.

### 2.4 Ca^2+^ concentration

The penis tissue of the rats was ground and lysed. The instructions provided with the Ca^2+^ concentration assay kit (S1063S, Shanghai Biyuntian Biotechnology Co., LTD, China) were followed. The absorbance at 575 nm was measured using an enzyme marker, and the sample Ca^2+^ concentration was calculated by plotting the standard curve.

### 2.5 Real-time quantitative polymerase chain reaction (RT-qPCR)

Total RNA was isolated from penile tissue with the TRIzol reagent (Thermo Fisher Scientific, United States). RNA concentration was quantified using a spectrophotometer (NanoDrop 2000C, Thermo Fisher Scientific, United States), and then reverse-transcription of RNA to produce cDNA (KR118, Beijing TIANGEN Biotech Co., LTD, China). RT-qPCR was performed using SYBR^®^ TB Green Premix Ex Taq. The primer sequences are as follows: RIP3 (sense, 5′-TCT​TAC​TGA​GAG​GAG​AGG​AAA GGA-3'; antisense, 5′-GAG​GGT​AAA​GTA​TGT​GGA​ATT​TGG-3′), MLKL (sense, 5′-AACTGA GCA​CGA​TTT​ATA​GAG​GAG​A-3'; antisense, 5′-GAG​CCT​CAC​TAT​TCC​AAC​ACT​T TC-3′), TRPM7 (sense, 5′-GTG​TTC​CTG​TGG​TGG​CTT​TG-3'; antisense, 5′-TCC​CTC​CTT​CCT​CTG​TCT GC-3′), β-Actin (sense, 5′-TGA​CAG​ACT​ACC​TCA​TGA​AGA​TCC-3'; antisense, 5′-GCAACAT AGC​ACA​GCT​TCT​CTT​TA-3′). All primers were designed NCBI Primer Blast and synthesized by Shanghai Sangon Biological Co., LTD, China. The method of 2^−ΔΔCT^ was used to perform quantitative analysis. All results were normalized using the β-Actin gene.

### 2.6 Western blotting

Penile tissue was homogenized in cell lysis buffer containing protease inhibitors. After the quantification of protein by the BCA assay (PC0020, Beijing Solaibao Technology Co., LTD, China), total of 20 μg of protein lysate for each sample would be subjected to electrophoresis, transmembrane and blocked the membranes, respectively. The membranes were incubated with the primary antibody and β-Actin (1:6000, AF7018, Affinity, Cincinnati, OH, United States), RIP3 (1:1000, AF7942, Affinity), p-RIP3 (1:1000, ab255705, Abcam), MLKL (1:2000, 66675-1-Ig, Proteintech, Wuhan, China), p-MLKL (1:1000, Affinity), TRPM7 (1:1000, DF7513, Affinity), eNOS (1:2000, AF0096, Affinity), ET-1 (1:1000, DF6125, Affinity), TNF-α (1:1000, AF7014, Affinity), IL-6 (1:2000, DF6087, Affinity), overnight, at 4°C. The membranes were then washed with TBST and treated with secondary antibody, including goat anti-mouse IgG (1:10,000, ZB2305, ZSGB-BIO, Beijing, China), and goat anti-rabbit IgG (1:10,000; ZB2301, ZSGB-BIO). The blots were visualized with an enhanced chemiluminescence system. The ImageJ was employed to quantify the intensity of the band, with the results subsequently normalised to the β-actin signal intensity.

### 2.7 Double immunofluorescence

The penile paraffin sections underwent routine dewaxing and rehydration procedures. The tissue sections were immersed in the antigen repair solution at 95°C for 30 min and allowed to cool naturally. Subsequently, the sections were incubated with primary antibodies, RIP3 (1:100, AF7942, Affinity, Cincinnati, OH, United States), MLKL (1:200, 66675-1-Ig, Proteintec), TRPM7 (1:50, DF7513, Affinity). Images were obtained and analysed using an inverted fluorescence microscope, and the mean fluorescence intensities of RIP3, MLKL, and TRPM7 were quantified using the ImageJ.

### 2.8 Statistical analyses

Data were statistically analyzed and normality tests using SPSS version 23.0 (IBM, Armonk, United States), and presented as mean ± standard deviation ( $\bar{x}\pm s$ ). Groups of data were compared using a two-tailed unpaired Student’s t*-*test and one-way of variance (ANOVA), followed by a Tukey-Kramer post-hoc analysis. Statistical significance was set at *p* < 0.05.

## 3 Results

### 3.1 Increased tissue damage and cell death in penile tissue of DMED rats

**FIGURE 1:**
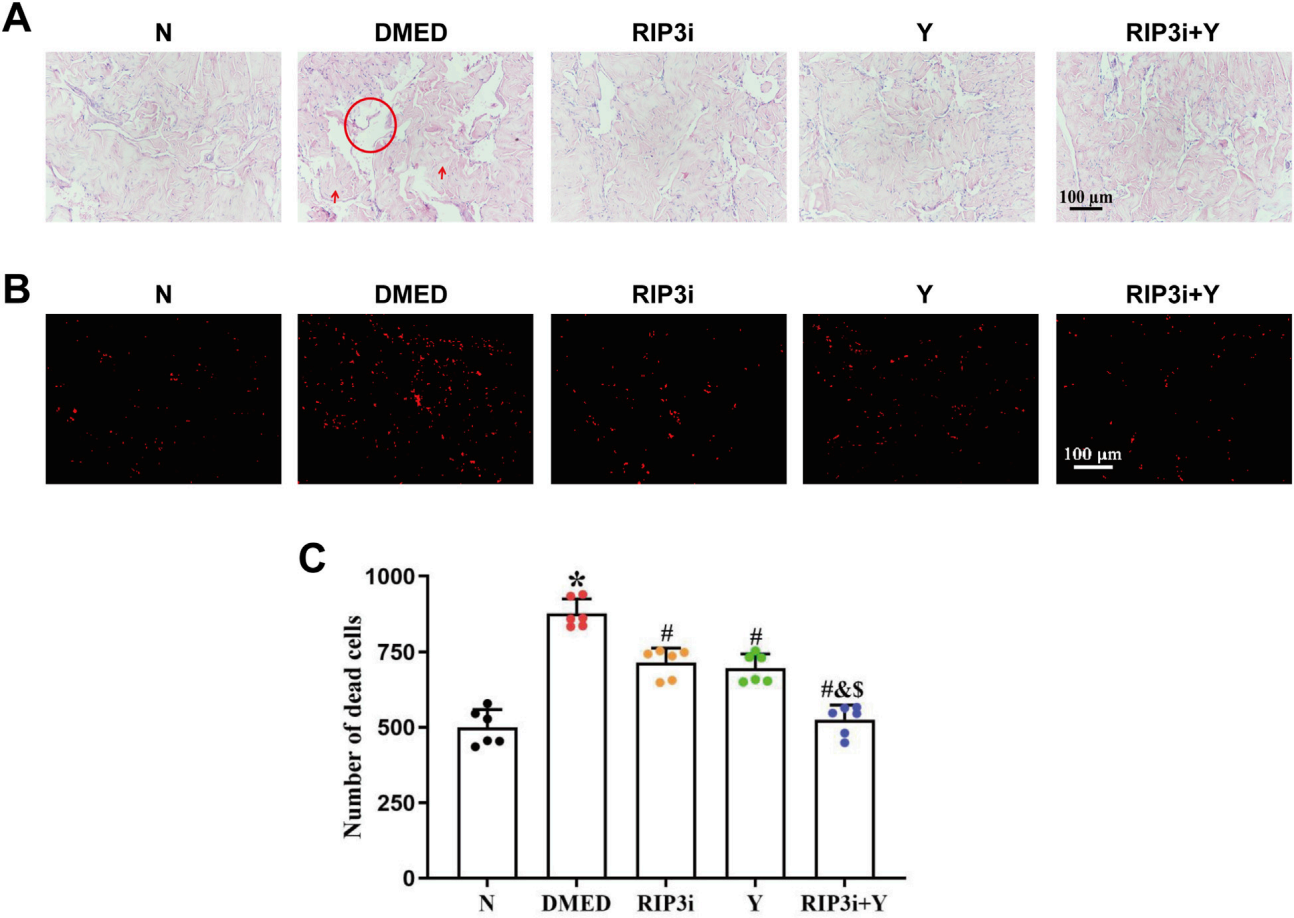
Morphological changes of rat penis tissue. **(A)** Representative images of HE staining (magnification, × 200). Scale bars = 100 μm. High glucose-induced enlargement of the cavernous sinus cavity of the penis (circle) and increase in the quantity of collagen fibres (arrows), while these improve after the administration of the RIP3 inhibitor or Yimusake. **(B)** Propidium iodide staining used to determine the number of cell disintegration and death among the group (magnification, × 200). Scale bars = 100 μm. **(C)** Quantitative analysis of the propidium iodide-positive cells. All data are presented as the mean ± SEM (n = 6). ^*^ *p* < 0.05 in comparison with the N group. ^#^ *p* < 0.05 in comparison with the DMED group. ^&^ *p* < 0.05 in comparison with the RIP3i group. ^$^ *p* < 0.05 in comparison with the Y group.

We constructed diabetic rats by intraperitoneal injection of streptozotocin (45 mg/kg), and the DMED rat model was successfully screened by random blood glucose, APO experiments, and paired experiments (the results are not included in the article). Preliminary experiments by our research team showed that the key necroptosis-associated indicators RIP1 and RIP3 were significantly upregulated in the penile tissues of ED rats (Yang et al., 2024), suggesting that the necroptotic pathway may play a regulatory role in the progression of ED. To further investigate the precise pathological mechanism of DMED rats, this study used the RIP3 inhibitor (GSK872) to treat DMED rats, and Yimusake, a traditional medicine commonly used for ED, as a positive control. After 2 weeks of treatment, we observed the changes in the pathological structure of the penile tissue using HE staining. The results are shown in Figure 1A, the penile tissue from group N exhibited normal morphology, abundant cavernous sinuses, and intact endothelial structure. Conversely, compared with the N group, the DMED group displayed a reduction in cavernous sinus count, enlargement of the cavernous sinus cavity, decreased density of smooth muscle cells and endothelial cells, as well as an increase in collagen fiber quantity. However, following 2 weeks of treatment with the RIP3 inhibitor and Yimusake, a significant improvement in the cavernous tissue damage and a reduction in the number of cavernous sinus cavities was observed in rats in the RIP3i group and the Y group, with the greatest reduction observed in the RIP3i + Y group of rats. Furthermore, we conducted IP experiments to further explore the damage of cavernous tissue in rats. The results showed a significant increase in the number of dead cells in the penile tissue of DMED rats (*p* < 0.05; Figures 1B, C), indicating that high glucose can promote morphological changes and increase the cell death rate in the penile. Furthermore, the effect of treatment with the RIP3 inhibitor or/and Yimusake was consistent with the results of the HE staining.

### 3.2 High levels of the RIP3/MLKL/TRPM7 necroptotic pathway in penile tissue of DMED rats

**FIGURE 2:**
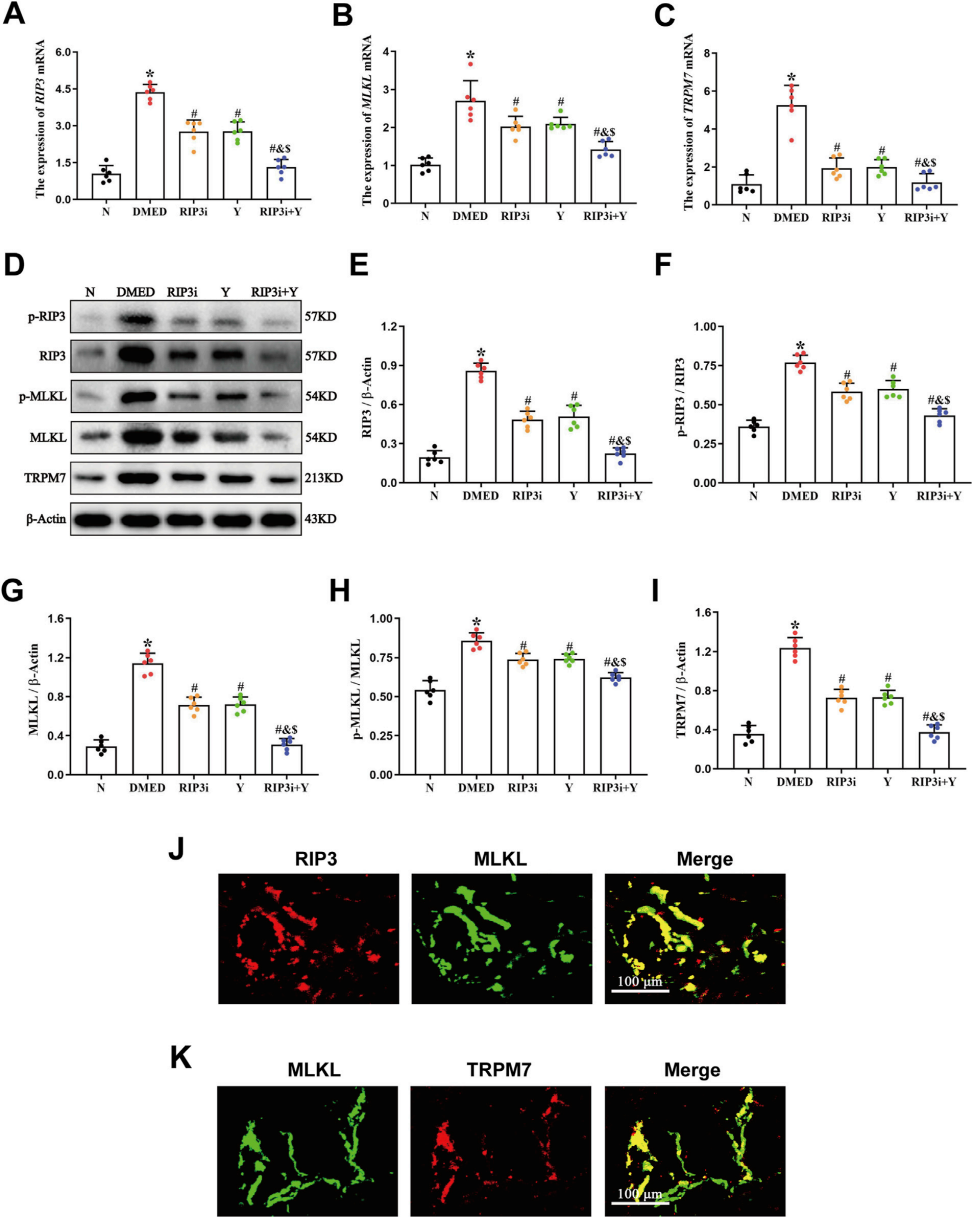
Expression of necroptosis-associated proteins in penile tissue of rats. RT-qPCR was used to determine the expression of **(A)** *RIP3*, **(B)** *MLKL*, and **(C)** *TRPM7* mRNA in five groups of penile tissues. **(D)** The expression of the proteins RIP3, p-RIP3, MLKL, p-MLKL, and TRPM7 in rat penile tissues of each group was determined by Western blotting. Quantitative analysis of the expression of **(E)** RIP3, **(F)** p-RIP3, **(G)** MLKL, **(H)** p-MLKL and **(I)** TRPM7. Double immunofluorescence of RIP3 and MLKL **(J)**, MLKL and TRPM7 **(K)** in rat penile tissue. Red fluorescence indicated RIP3 or TRPM7 signals, green fluorescence represented MLKL signals. Scale bars: 100 µm. Merge: all images in the row. All data are presented as the mean ± SEM (n = 6). *p < 0.05 in comparison with the N group. ^#^ *p* < 0.05 in comparison with the DMED group. ^&^ *p* < 0.05 in comparison with the RIP3i group. ^$^ *p* < 0.05 in comparison with the Y group.

RT-qPCR and Western blot experiments were performed to measure the levels of mRNA expression and protein levels of necroptosis-associated indicators, RIP3, MLKL, and TRPM7, in rat penile tissue. Compared to the N group, the expression of RIP3, MLKL and TRPM7 mRNA and protein levels increased significantly in the DMED group (all *p* < 0.05; Figures 2A–I). After treatment with RIP3 inhibitor and Yimusake, the expression levels of RIP3, MLKL, and TRPM7 were reduced in all treatment groups compared to the DMED group (all *p* < 0.05). The reduction was particularly significant in the RIP3i + Y group (*p* < 0.05). In particular, p-RIP3 and p-MLKL levels were elevated in DMED and significantly reduced under treatment conditions (all *p* < 0.05). Moreover, the changes in p-RIP3 and p-MLKL levels in the penile tissues of the groups are identical as above. Therefore, high glucose may lead to impaired penile function in DMED rats by activating the RIP3/MLKL/TRPM7 necroptotic pathway. Using double immunofluorescence staining, we observed the intensity and distribution of staining for RIP3 and MLKL in penile tissues, as well as for MLKL and TRPM7. This further verifies the above conclusion (Figures 2J, K).

### 3.3 Inhibition of RIP3 reduces Ca^2+^ and inflammation levels in the corpus cavernosum of DMED rats

**FIGURE 3:**
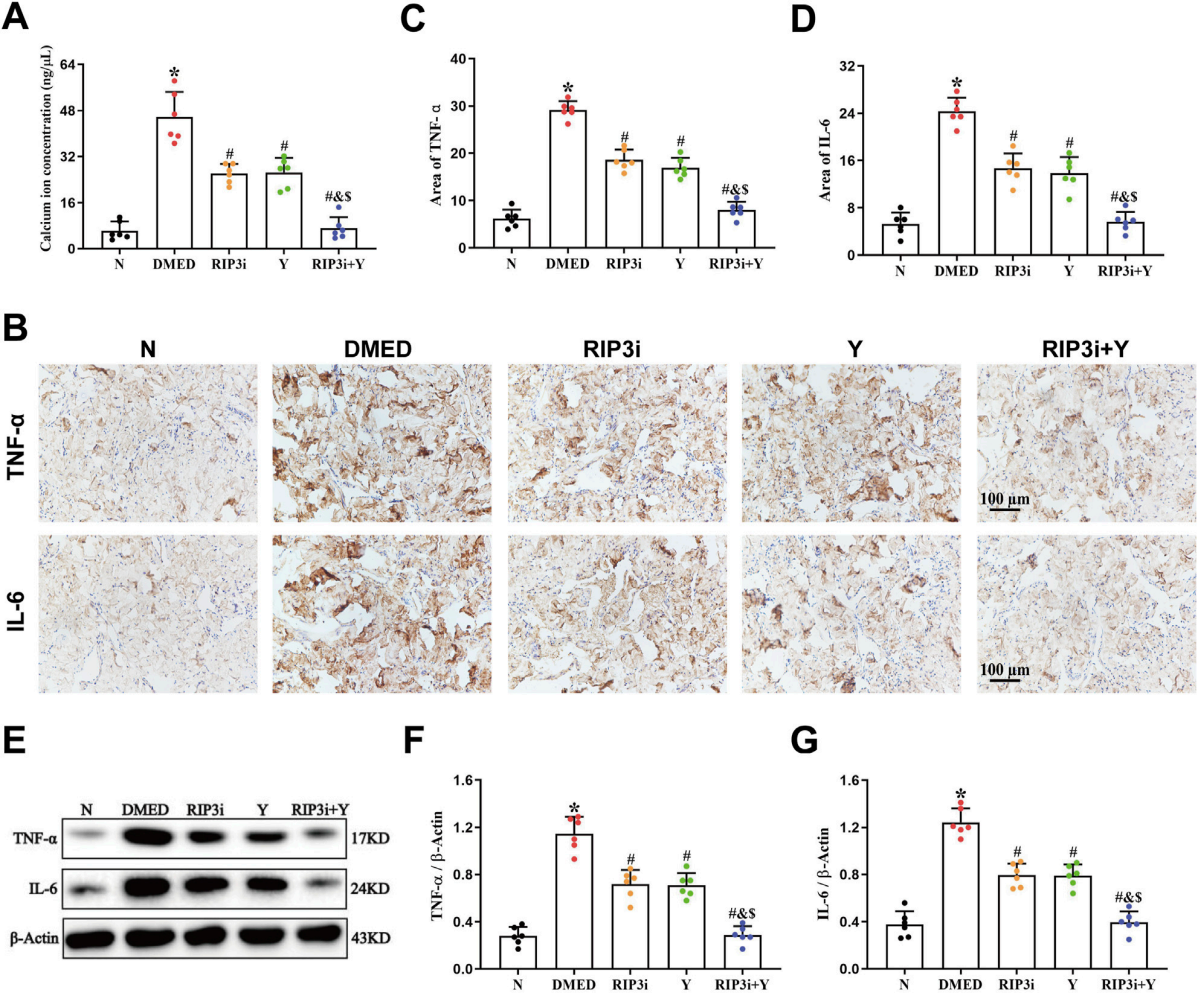
Alterations in the Ca^2+^ concentration and inflammation of rat penile tissue. **(A)** Comparisons of Ca^2+^ concentration in the penis tissue among the group. **(B)** Immunohistochemistry was used to determine the levels of TNF-α and IL-6 proteins of each group. Area of **(C)** TNF-α and **(D)** IL-6. **(E)** Western blotting was used to determine the expression of TNF-α and IL-6 proteins of each group. Quantitative analysis of the expression of **(F)** TNF-α and **(G)** IL-6. All data are presented as the mean ± SEM (n = 6). *p < 0.05 in comparison with the N group. ^#^ *p* < 0.05 in comparison with the DMED group. ^&^ *p* < 0.05 in comparison with the RIP3i group. ^$^ *p* < 0.05 in comparison with the Y group.

TRPM7 is a membrane protein that has both ion channel and kinase activities. It has been extensively studied in cardiomyocytes, where its activation leads to Ca^2+^ inward flow, resulting in impaired cell function and even death. To further clarify the function of TRPM7 in DMED, we also analyzed the changes in Ca^2+^ levels in rat corpus cavernosum tissues. This study revealed a significant increase in the concentration of Ca^2+^ of the DMED group compared to the N group (all *p* < 0.05; Figure 3A). The concentrations of Ca^2+^ were observed to be lower in the RIP3i and Y groups in comparison to the DMED group, and the RIP3i + Y group showing the most pronounced improvement (all *p* < 0.05). Furthermore, another finding indicated that the cell death rate in the corpus cavernosum was significantly increased by high glucose induction, which is consistent with the changes in Ca^2+^ concentration (*p* < 0.05; Figure 3A), and inhibition of RIP3 was effective in improving cell death in the corpus cavernosum of rats. Our study provides further evidence for the activation of the RIP3/MLKL/TRPM7 pathway in rat corpus cavernosum tissues under high glucose conditions. It also suggests that the upregulation of necroptosis may be a significant factor contributing to penile tissue injury in rats.

In addition, necroptosis is closely associated with inflammation, and the release of cellular debris and organelles from disintegrating dead cells plays a crucial role in promoting heightened levels of inflammation. We also investigated the level of inflammation in the rat penile. Our findings revealed that the expression levels of both TNF-α and IL-6 were significantly elevated in the DMED group compared to the N group (all *p* < 0.05; Figures 3B–G). However, upon inhibition of RIP3, there was a significant downregulation observed in the expression levels of TNF-α and IL-6, similar to those observed in the Y group (all *p* < 0.05). Furthermore, the RIP3i + Y group exhibited a more pronounced reduction in inflammation compared to both RIP3i and Y groups alone (all *p* < 0.05).

### 3.4 Inhibition of RIP3 significantly improves erectile function of DMED rats

**FIGURE 4:**
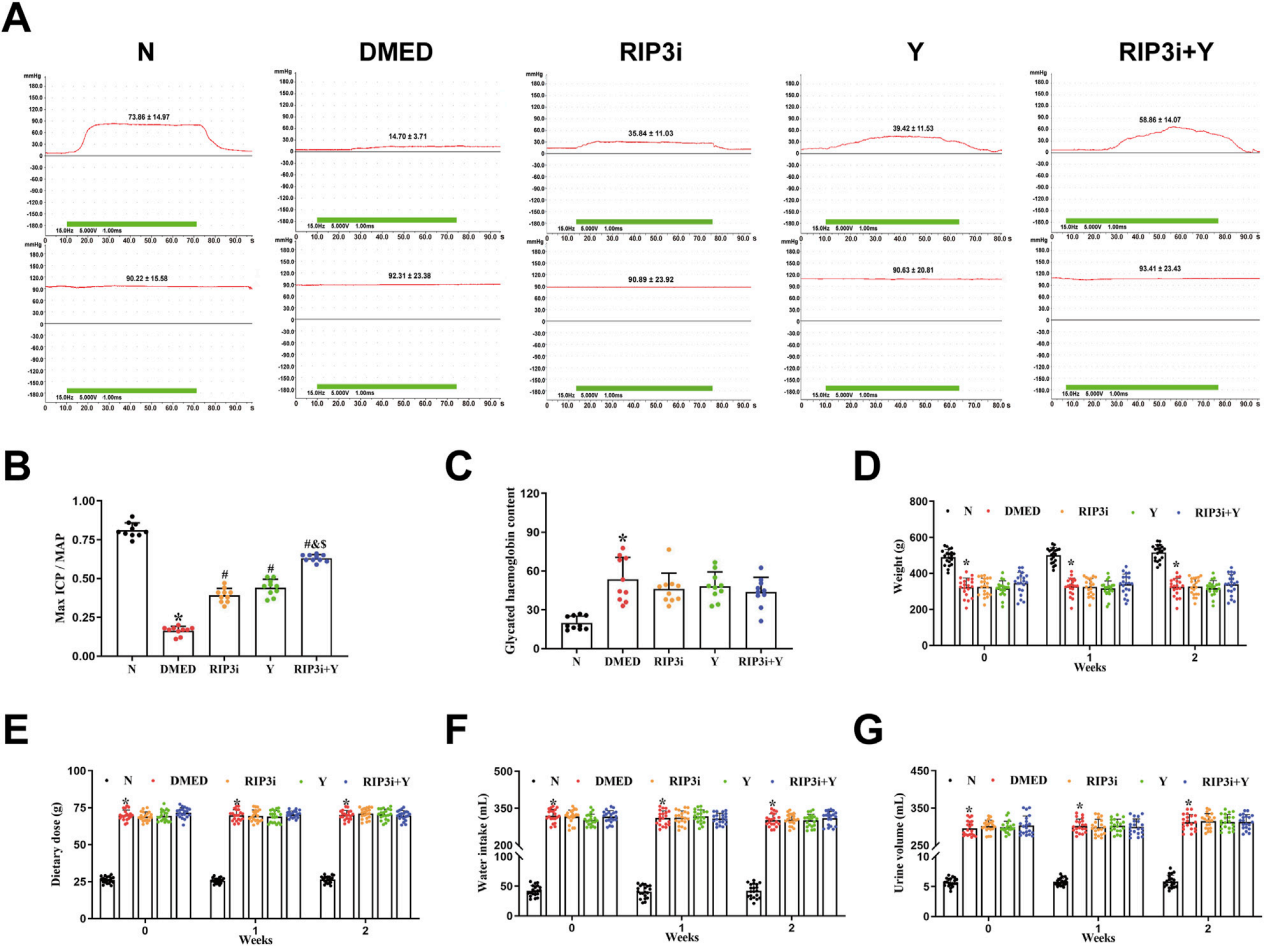
Alterations in penile erection, metabolic and physiological parameters of rats during treatment. **(A)** The ICP and MAP in different groups under electrical stimulation (15 Hz, 7.5 V, 1 min). **(B)** The Max ICP/MAP of rats. **(C)** Changes in glycated haemoglobin levels in rats. **(D)** The weight of rats. **(E)** The diet of rats. **(F)** The water intake of rats. **(G)** The urine volume of rats. All data are presented as the mean ± SEM (n = 6). *p < 0.05 in comparison with the N group. ^#^ *p* < 0.05 in comparison with the DMED group. ^&^ *p* < 0.05 in comparison with the RIP3i group. ^$^ *p* < 0.05 in comparison with the Y group.

To investigate changes in penile erection of rats during treatment, we measured arterial pressure and ICP in each rat group after 2 weeks of treatment. The results revealed that, while maintaining relatively constant MAP, the DMED group exhibited significantly reduced maximum ICP/MAP compared to N group (*p* < 0.05; Figures 4A, B). In contrast, the maximum ICP/MAP of rats were significantly improved in comparison with the DMED group after treatment with RIP3 inhibitor and Yimusake, respectively (all *p* < 0.05). Notably, the RIP3i + Y group of rats showed even more pronounced significant (*p* < 0.05). Treatment with RIP3 inhibitors resulted in a partial alleviation of the destructive effects observed in penile tissue of DMED rats. Furthermore, the ameliorative influence of RIP3 inhibitors was enhanced by Yimusake. Moreover, glycated haemoglobin, a pivotal marker for evaluating blood glucose levels, was observed to be significantly elevated in the DMED group (*p* < 0.05, Figure 4C), and there was no significant improvement observed after treatment with RIP3 inhibitor and Yimusake. To further clarify whether inhibition of penile necroptosis improves erectile function in rats by ameliorating the symptoms of diabetes, we examined typical clinical symptoms of diabetes, “three more and one less”. Following an 8-week induction period, the DMED group demonstrated elevated metabolic indices, including water intake, food intake, and urine output, in addition to elevated blood glucose levels. However, they exhibited a notable reduction in body weight. Treatment with the RIP3 inhibitor and Yimusake for 2 weeks did not improve the diabetic symptoms mentioned above (all *p* < 0.05, Figures 4D–G). Thus, treatment with the RIP3 inhibitors partially improved the function of rat penile tissues, again demonstrating the significant role of RIP3-mediated necroptosis in regulating the course of DMED disease, and that inhibiting RIP3-mediated necroptosis does not improve diabetic symptoms in DMED rats.

### 3.5 Inhibition of RIP3 alleviates the fibrosis in the CCSMCs of DMED rats

**FIGURE 5:**
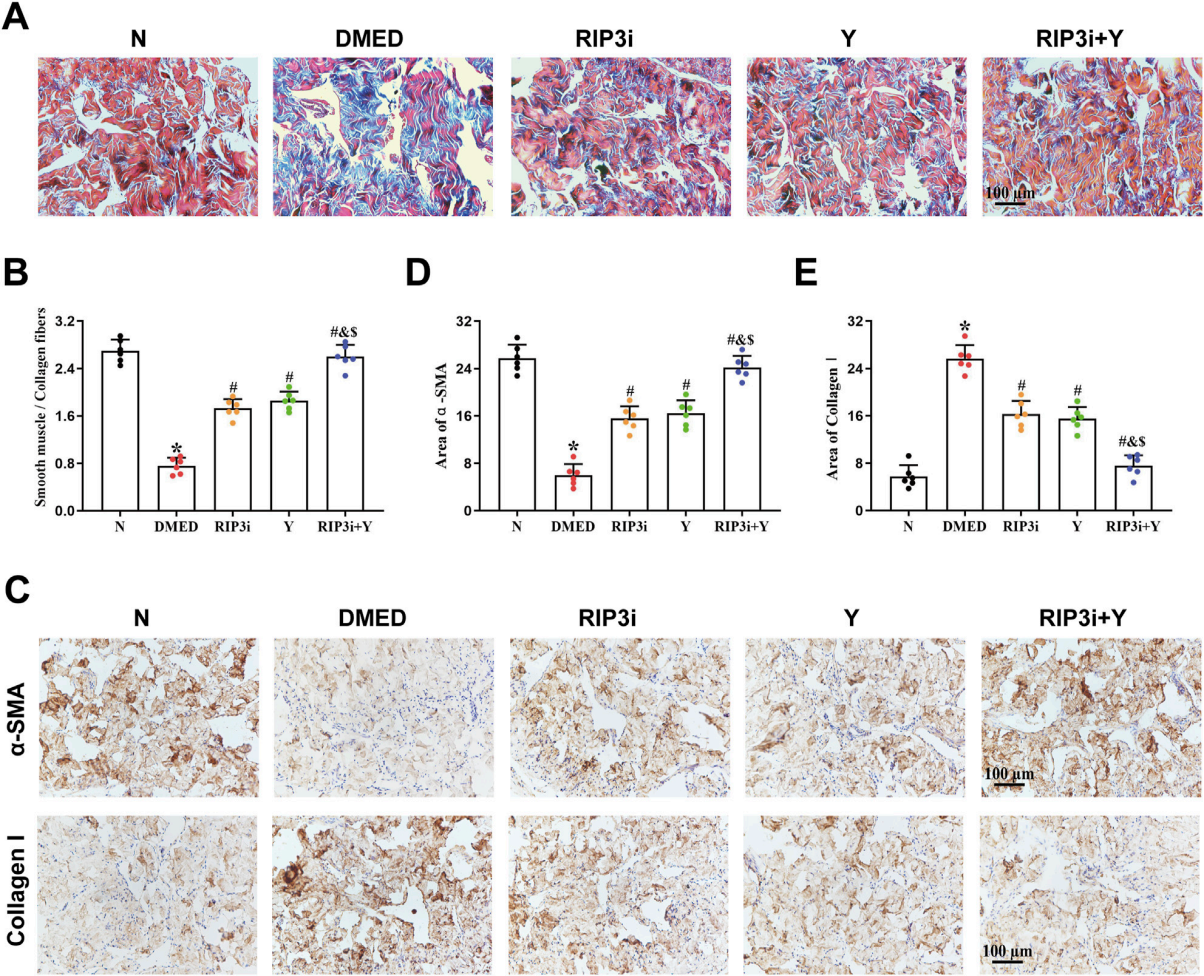
Morphological changes of rat penis tissue. **(A)** Representative images of Masson trichrome staining (magnification, × 200). The smooth muscle was stained red, while the collagen fibers were stained blue. **(B)** Semi-quantitative analysis of the ratio of the smooth muscle to collagen fibers. **(C)** Immunohistochemistry was used to determine the levels of α-SMA and Collagen I proteins of each groups. Area of **(D)** α-SMA and **(E)** Collagen I. Scale bars = 100 μm. All data are presented as the mean ± SEM (n = 6). *p < 0.05 in comparison with the N group. ^#^ *p* < 0.05 in comparison with the DMED group. ^&^ *p* < 0.05 in comparison with the RIP3i group. ^$^ *p* < 0.05 in comparison with the Y group.

Fibrosis of CCSMCs, key effector cells in corpus cavernosum tissue, is one of the main causes of organic ED. Masson trichrome staining was used to further clarify the underlying cause of reduced erectile function in DMED rats. The findings indicated a reduction in muscle fibre content within the corpus cavernosum tissue of the DMED group, accompanied by numerous breaks and a significantly lower smooth muscle/collagen fibre ratio in comparison to the N group (all *p* < 0.05; Figures 5A, B). Following treatment, the histopathological damage to the corpus cavernosum in both the RIP3i and Y groups showed varying degrees of improvement compared to the DMED group. And the restoration was observed in both muscle and collagen fibers, with a significant increase in the ratio of smooth muscle to collagen fibers. Moreover, the expression of α-SMA and Collagen I in the corpus cavernosum was evaluated through immunohistochemical analysis. The results showed that a significant decrease in α-SMA expression within the DMED group, while Collagen I expression was upregulated (all *p* < 0.05; Figures 5C–E), indicating an increase in the fibrosis of the CCSMCs. Notably, there was a significant improvement in fibrosis within the CCSMCs in all groups after treatment with the RIP3 inhibitors and Yimusake (all *p* < 0.05), consistent with the results of the Masson staining. Therefore, inhibition of the necroptosis pathway, which is mediated by RIP3, may have therapeutic effects on DMED by improving fibrosis in CCSMCs of rats.

### 3.6 Inhibition of RIP3 alleviates endothelial impairment of DMED rats

**FIGURE 6:**
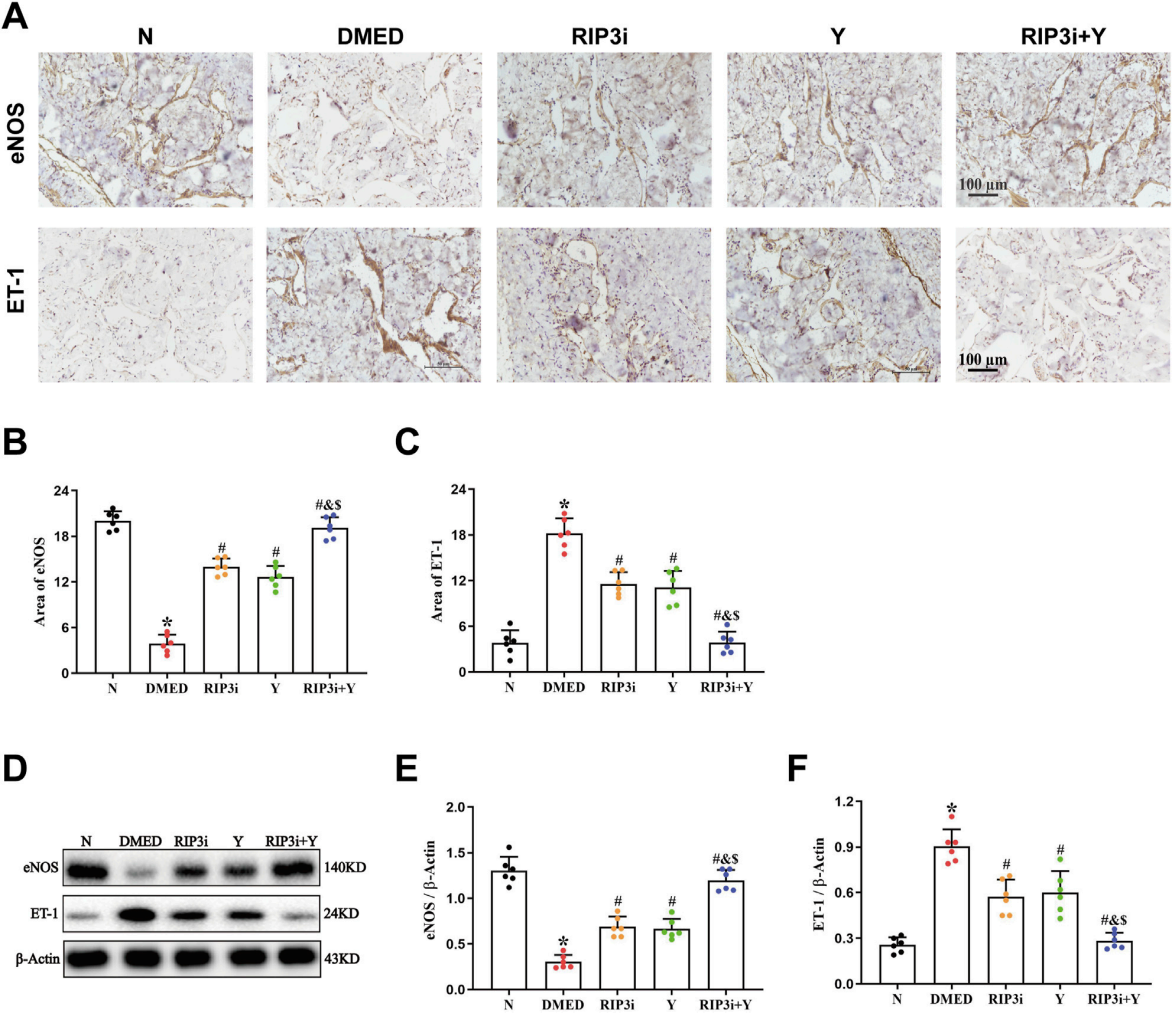
Changes in eNOS and ET-1 in the corpus cavernosum tissues of rats. **(A)** Immunohistochemistry was used to determine the levels of α-SMA and Collagen I proteins of each group (magnification, × 200). Area of **(B)** eNOS and **(C)** ET-1. **(D)** Western blotting was used to determine the expression of the eNOS and ET-1 proteins of each group. Quantitative analysis of the expression of **(E)** α-SMA and **(F)** Collagen I. Scale bars = 100 μm. All data are presented as the mean ± SEM (n = 6). *p < 0.05 in comparison with the N group. ^#^ *p* < 0.05 in comparison with the DMED group. ^&^ *p* < 0.05 in comparison with the RIP3i group. ^$^ *p* < 0.05 in comparison with the Y group.

The results of the HE staining showed that the density of endothelial cells was also altered in the penile tissue of DMED rats (Figure 1A), suggesting that endothelial dysfunction also be a cause of reduced penile function. To further verify the above results, we detected the expression of eNOS and ET-1, which are specific markers for endothelial cells, in the corpus cavernosum tissues of rats. It was found that the level of eNOS was significantly reduced and ET-1 increased in the penile tissue of the DMED group compared to the N group (all *p* < 0.05; Figures 6A–F). In the cavernous tissue, treatment with RIP3 inhibitors and Yimusake resulted in upregulation of eNOS and downregulation of ET-1. The improvement was more pronounced in the RIP3i + Y group (*p* < 0.05). This result suggests that high glucose may lead to endothelial dysfunction of the corpus cavernosum through activation of RIP3-mediated necroptosis, triggering DMED in rats.

## 4 Discussion

Necroptosis is a type of cell death that triggers an inflammatory response due to cell breakdown (Chen et al., 2022). Inflammatory factors, such as TNF-α and IFN-γ, are also crucial in inducing necroptosis (Moriwaki and Chan, 2017; Zhan et al., 2021). This suggests that inflammation and necroptosis are mutually causative. The presence of high levels of inflammation in patients with DMED is well documented (Yang et al., 2022). Therefore, necroptosis may play a crucial regulatory role in the course of DMED. Our previous study showed that RIP3, the key upstream signalling factors for necroptosis, was significantly upregulated in the penile tissues of rats with ED (Yang et al., 2024). However, it is unclear how RIP3-mediated necroptosis affects DMED. The study evaluated the effects of RIP3-mediated necroptosis on rat penile function by inhibiting RIP3 with specific inhibitors. We found that the RIP3-specific inhibitor, GSK872 (Guo et al., 2019), significantly reduced fibrosis and alleviated the death and functional impairment of CCSMCs, thereby improving penile erection in DMED rats. Additionally, RIP3 inhibition decreased inflammation levels in penile tissues, which further affected the expression of key indicator proteins for necroptosis, thereby improving high glucose-induced tissue damage. Our findings indicate that the mechanism of action of RIP3 on male rat fertility may be related to the activation of its downstream MLKL/TRPM7 signalling pathway.

The RIP3/MLKL pathway is a well-established necroptotic pathway that has been extensively studied and demonstrated in a variety of cardiovascular diseases (Hu et al., 2022; Lewno et al., 2021; Zhe-Wei et al., 2018). However, there are many controversies regarding the downstream signalling mechanisms of MLKL. TRPM7 is a membrane protein that functions as both a divalent cation channel and a protein kinase (Suzuki et al., 2020). It plays a crucial role in various pathophysiological processes *in vivo*, including ion transport and homeostasis of Ca^2+^ and Mg^2+^, as well as induction of cell death (Caglar et al., 2024). Cai et al. discovered that TRPM7 expression increased in human embryonic kidney cells overexpressing MLKL (Cai et al., 2014), suggests that TRPM7-mediated Ca^2+^ inward flow is involved in necroptotic plasma membrane rupture. The RIP3/MLKL/TRPM7 necroptotic pathway is being progressively explored (Shi et al., 2021). Hypoxia has been found to worsen endothelial cell injury in rat cardiac microvessels through the activated RIP3/MLKL/TRPM7 pathway (Bai et al., 2019). The pathway is also a significant factor in the aggravation of cardiac fibrosis in rat cardiomyocytes under high glucose conditions (Kang et al., 2020). Furthermore, activation of the RIP3/MLKL/TRPM7 pathway also worsens inflammation in brain tissue of rats suffering from traumatic brain injury (Ni et al., 2019). Thus, the RIP3/MLKL/TRPM7 necroptotic pathway is closely associated with vascular endothelial damage, tissue fibrosis, and high levels of inflammation, which are also common pathological symptoms of DMED (Li et al., 2024). However, there is currently no clear report on the RIP3/MLKL/TRPM7 in DMED. Our study found that the expression levels of RIP3, MLKL and TRPM7 in penile tissues of DMED rats were closely correlated with blood glucose (Figure 2). Moreover, inhibition of RIP3 resulted in a downregulation of expression for all three proteins. Therefore, It is suggested that the pathogenesis of DMED in rats may be related to activation of the RIP3/MLKL/TRPM7 necroptotic pathway. Additionally, our analysis of Ca^2+^ in penile tissue revealed that the transport of this ion was disrupted (Figure 3A). This increase is the main cause of cell membrane rupture, which triggering cell disintegration and death. This was accompanied by a significant improvement in sexual function (Figure 4). The results further substantiate the importance of the RIP3/MLKL/TRPM7 necroptotic pathway in the pathogenesis of sexual impairment in male diabetic rats. This provides a novel avenue for a comprehensive exploration of the pathophysiological mechanisms of DMED.

The process of penile erection is based on diastole and contraction of the cavernous smooth muscle, which is largely attributed to the key effector cells, CCSMCs (Qinyu et al., 2021; Yilmaz-Oral et al., 2024). To investigate the specific target of necroptosis occurrence, we examined penile tissue fibrosis in rats. Consistent with the results of existing studies (Gur and Hellstrom, 2020; Nishimatsu et al., 2013; Sun et al., 2023), we have shown that high glucose not only promotes fibrosis of CCSMCs and impairs their diastolic and contractile functions (Figure 5), but more importantly, it significantly reduces the number of CCSMCs (Figure 1). Indicates that DMED in rats is strongly associated with fibrosis and death of CCSMCs. Furthermore, it is important to note that CCSMCs do not function independently, and their diastolic and contractile functions are also regulated by NO synthesized by endothelial cells (Chen et al., 2023). Our findings revealed that diabetes-induced male reproductive injury also led to enlargement of the cavernous sinus cavities, reduction in endothelial cell count, and dysregulation of eNOS and ET-1 levels (Figures 1A, 6). These alterations exacerbate erectile dysfunction by triggering impairment in both diastolic and systolic functions of CCSMCs, consistent with findings reported by Alkan et al. (2017), Shahin et al. (2018), and Ma et al. (2020). To clarify the mechanisms of functional impairment and death of CCSMCs and endothelial cells, we treated rats with a RIP3 inhibitor. The results showed a significant improvement in erectile function, as well as a reduction in fibrosis of CCSMCs and endothelial dysfunction in rats (Figures 4–6). The present study thus provides corroborating evidence for the hypothesis that high glucose levels induce DMED in part by promoting fibrosis, cell death, and functional impairment of CCSMCs in penile tissues. Furthermore, endothelial dysfunction contributes to the exacerbation of these pathological processes.

In addition, the development of DMED is highly correlated with a significant and persistent increase in inflammation (Sun et al., 2023). In DMED rats, high glucose led to a significant increase in the expression of inflammation-related proteins, including TNF-α, IL-6, and IL-1β, accompanied by histopathological damage to the penis (Maiorino et al., 2018; Mao et al., 2023; Matsui et al., 2021). Our findings are consistent with the conclusion (Figure 3). Combined with our previous finding of significant activation of RIP3/MLKL/TRPM7 necroptotic pathways (Figure 2), it suggests that local and systemic inflammatory responses triggered by high glucose may be closely associated with necroptosis. And that necroptosis-induced upregulation of the inflammatory response may also be an important factor in further exacerbating cell damage or even death. In particular, inhibition of RIP3/MLKL/TRPM7 pathway was effective in reducing local inflammation in the rat penis. Dan Ke et al. have also suggested that RIP3 could be a drug target for controlling inflammation in the treatment of diabetic complications (Ke et al., 2023). Therefore, we not only confirmed the existence and significance of necroptosis in the penile tissue of DMED rats, but also further explained the cause of high levels of inflammation in these rats.

**FIGURE 7:**
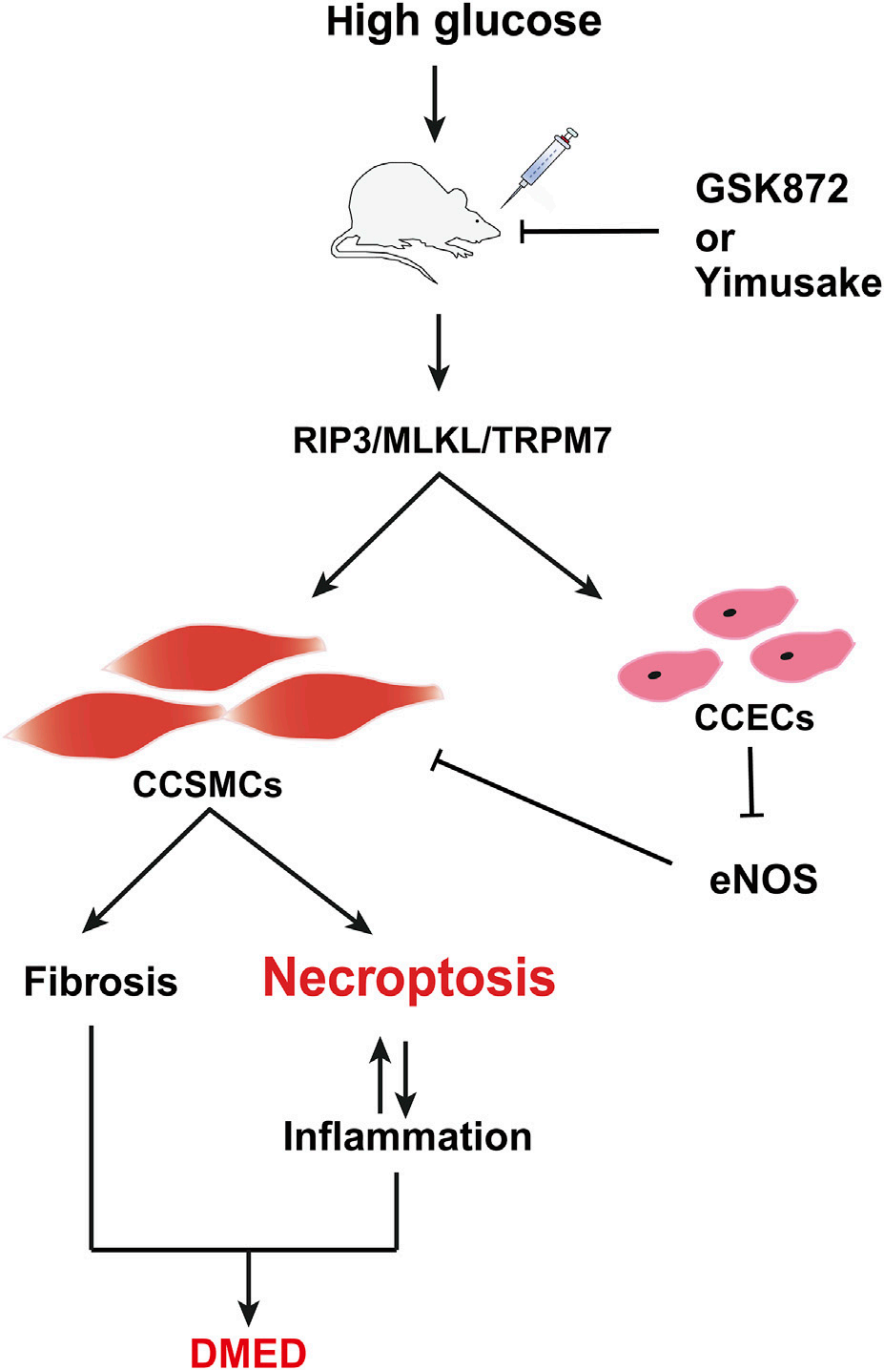
Potential mechanism of DMED induction by the RIP3/MLKL/TRPM7 necroptotic pathway. CCSMCs, Corpus cavernosum smooth muscle cells; CCECs, Corpus cavernosum endothelial cells.

The study also provides a valuable reference for refining the molecular mechanism of the Yimusake (in tablet form). The Yimusake is a traditional Chinese speciality herb that has been used for the treatment of ED and premature ejaculation (Jiang et al., 2019). It consists of a variety of herbs, including radices salep, ambergris, musk, nutmeg, Syzygium aromaticum, and other herbs (Zhai et al., 2020). Studies have shown that imusacum can facilitate penile erection by activating the NO/cGMP pathway, inhibiting endothelial damage, preventing smooth muscle fibrosis, etc. (Wang et al., 2017). The efficacy of the drug has been demonstrated in clinical applications, but the specific therapeutic mechanism has not been fully clarified. The analysis of the study results indicates that the therapeutic effect of Yimusake is comparable to that of RIP3 inhibitors. Both rescued high glucose-induced cellular damage by inhibiting RIP3 expression, which in turn downregulated the RIP3/MLKL/TRPM7 pathway and inflammation levels in penile tissue. Therefore, it is evident that these agents do not ameliorate DMED by down-regulating blood glucose levels (Figure 4). Suggests that the RIP3/MLKL/TRPM7 necroptotic pathway may affect penile tissue function mainly through local effects and does not improve diabetes. This reflects the complexity of the pathogenesis of high-glucose-induced ED and the limitations of monotherapy with RIP3 inhibitors or Yimusake. In particular, the study results showed that the combination of RIP3-specific inhibition and Yimusake was more effective than single drug treatment in reducing the level of necroptosis. It not only further demonstrated the importance of inhibiting necroptosis in DMED treatment, but also prove that Yimusake may reduce endothelial damage and smooth muscle fibrosis in the penile corpus cavernosum, lower inflammation levels by inhibiting the RIP3/MLKL/TRPM7 necroptotic pathway (Figure 7).

## 5 Conclusion

In conclusion, the inhibition of the RIP3/MLKL/TRPM7 necrotic pathway has the potential to be a therapeutic approach. This is because it not only improves the fibrosis and death of CCSMCs and maintains their contractile function, but also reduces the inflammatory response within the rat penile tissue (Figure 7). The combination of RIP3 inhibitors with the traditional Chinese medicine Yimusake is more effective. This provides an important reference for the efficient clinical treatment of DMED and a new research idea for the secondary development and clinical translation of Yimusake. Further validation of *in vitro* experiments is required to support the results of this study, which is the next stage of our research programme. Furthermore, diabetes mellitus is a systemic metabolic disease with a complex pathological mechanism that not only directly affects the penile erectile function, but may also trigger ED by mediating various organ functions and pathways, such as hormone levels, the gonadal axis, and testes. The study only investigated the overall therapeutic effects of different pharmacological interventions on DMED. However, the precise targets and mechanisms of action remained inadequately delineated, particularly with regard to the potential involvement of high glucose in ED through the promotion of necroptosis of CCECs. This represents a limitation of the present investigation. These issues will be the subject of further investigation.

## Data Availability

The original contributions presented in the study are included in the article/Supplementary Material, further inquiries can be directed to the corresponding author.
