# Supplementary figures and images for: Inhibition of the RIP3/MLKL/TRPM7 necroptotic pathway ameliorates diabetes mellitus-induced erectile dysfunction by reducing cell death, fibrosis, and inflammation
Source: Front Pharmacol. 2024 Sep 12;15:1436013. doi: 10.3389/fphar.2024.1436013 (PMC11424535; doi:10.3389/fphar.2024.1436013)

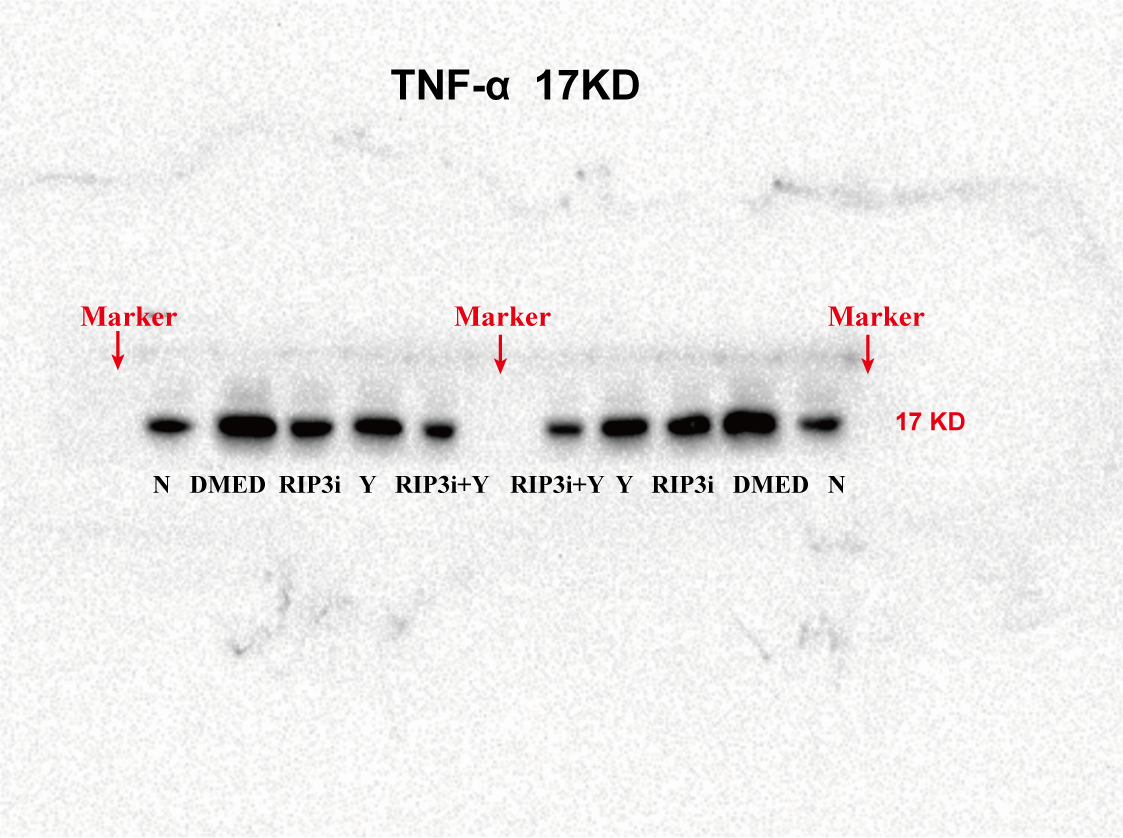

Supplement: Supplementary file 1 [file Image6.TIF]

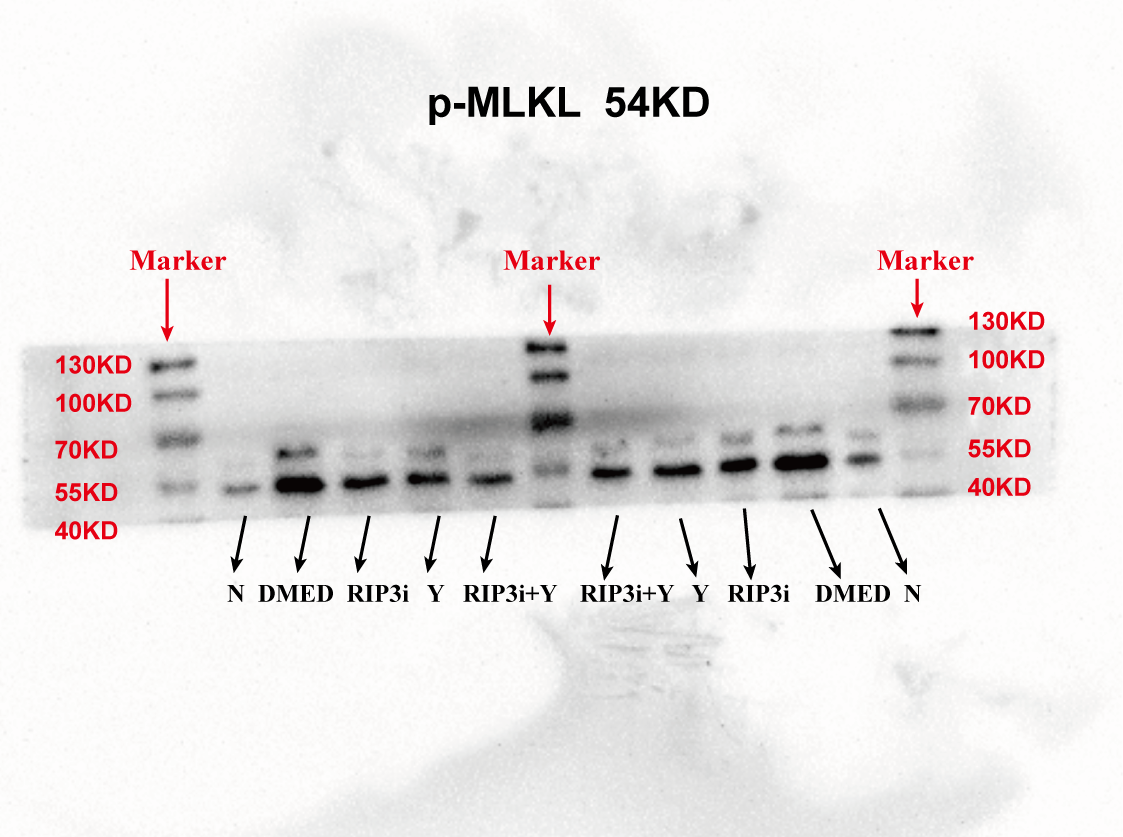

Supplement: Supplementary file 2 [file Image3.TIF]

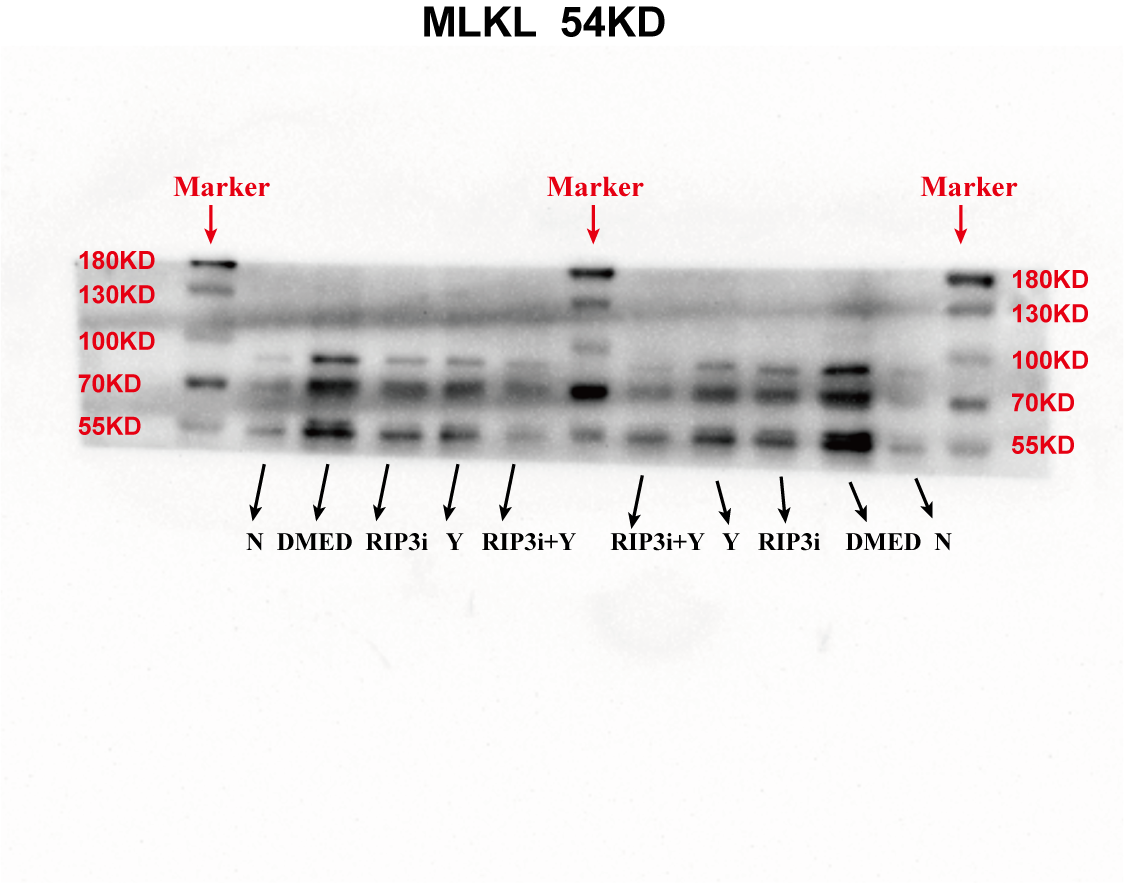

Supplement: Supplementary file 3 [file Image4.TIF]

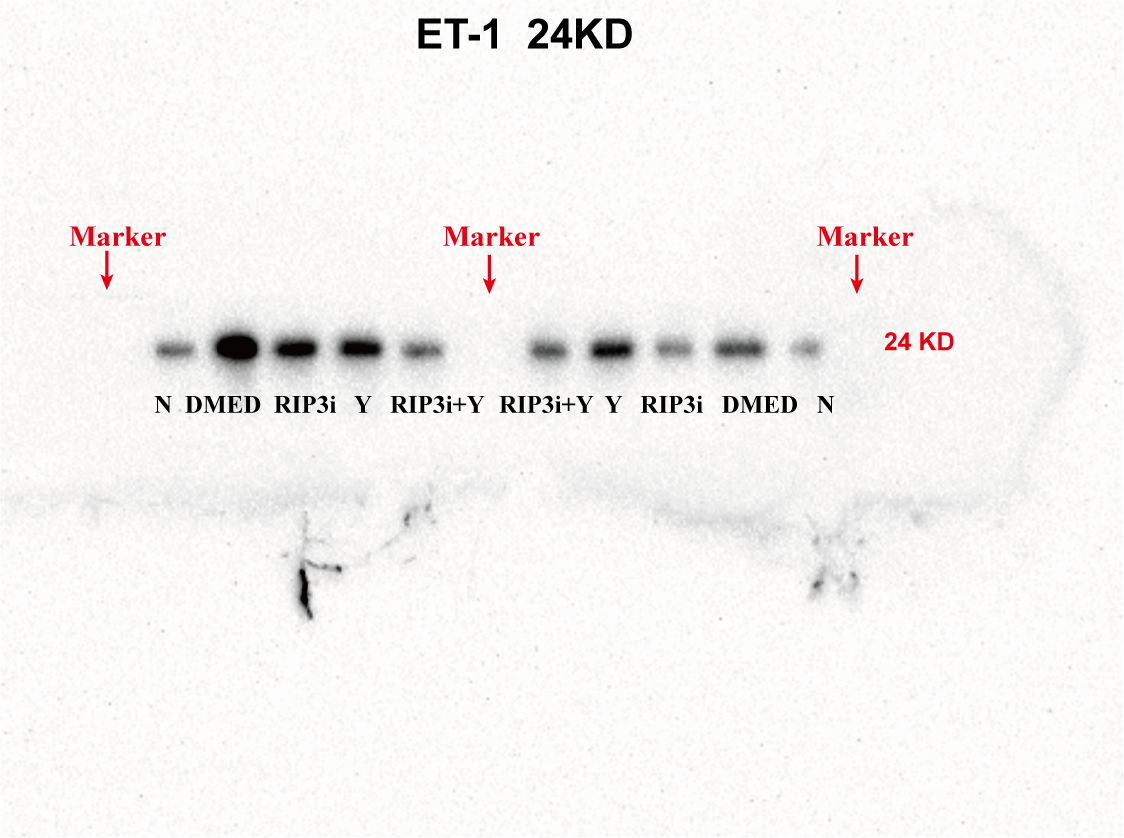

Supplement: Supplementary file 4 [file Image9.TIF]

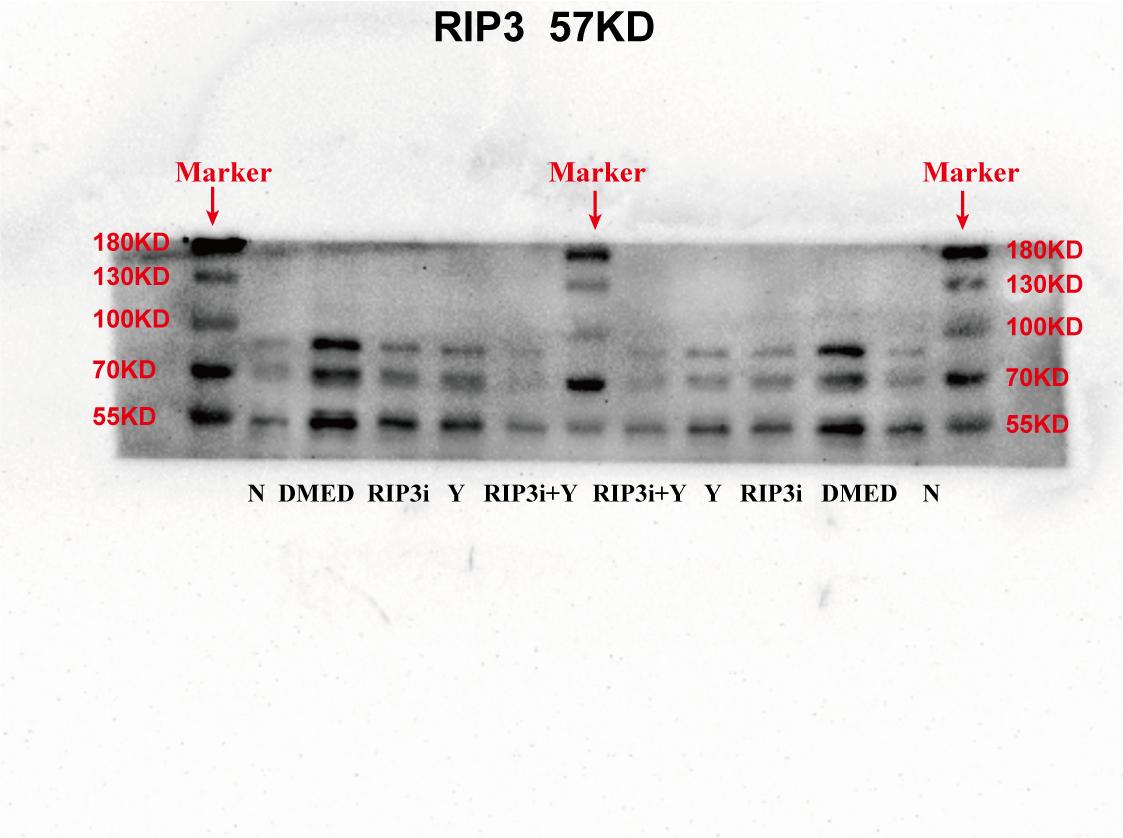

Supplement: Supplementary file 5 [file Image2.TIF]

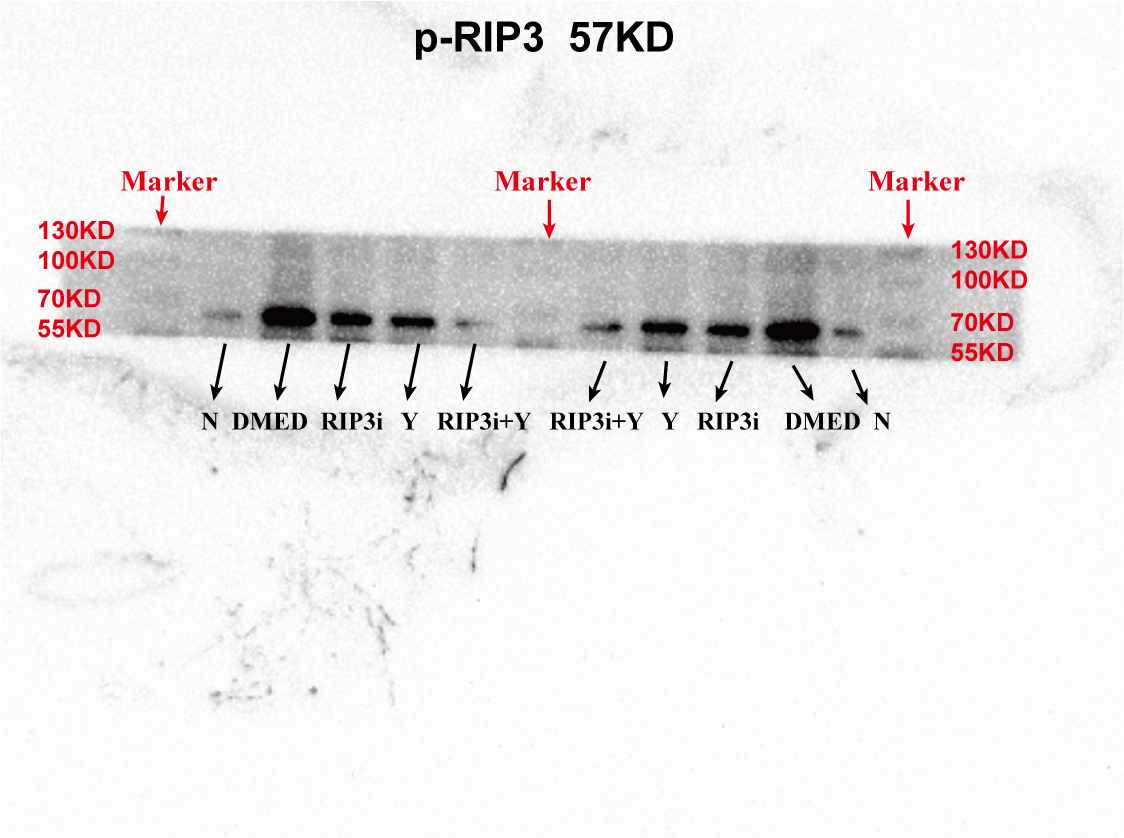

Supplement: Supplementary file 6 [file Image1.TIF]

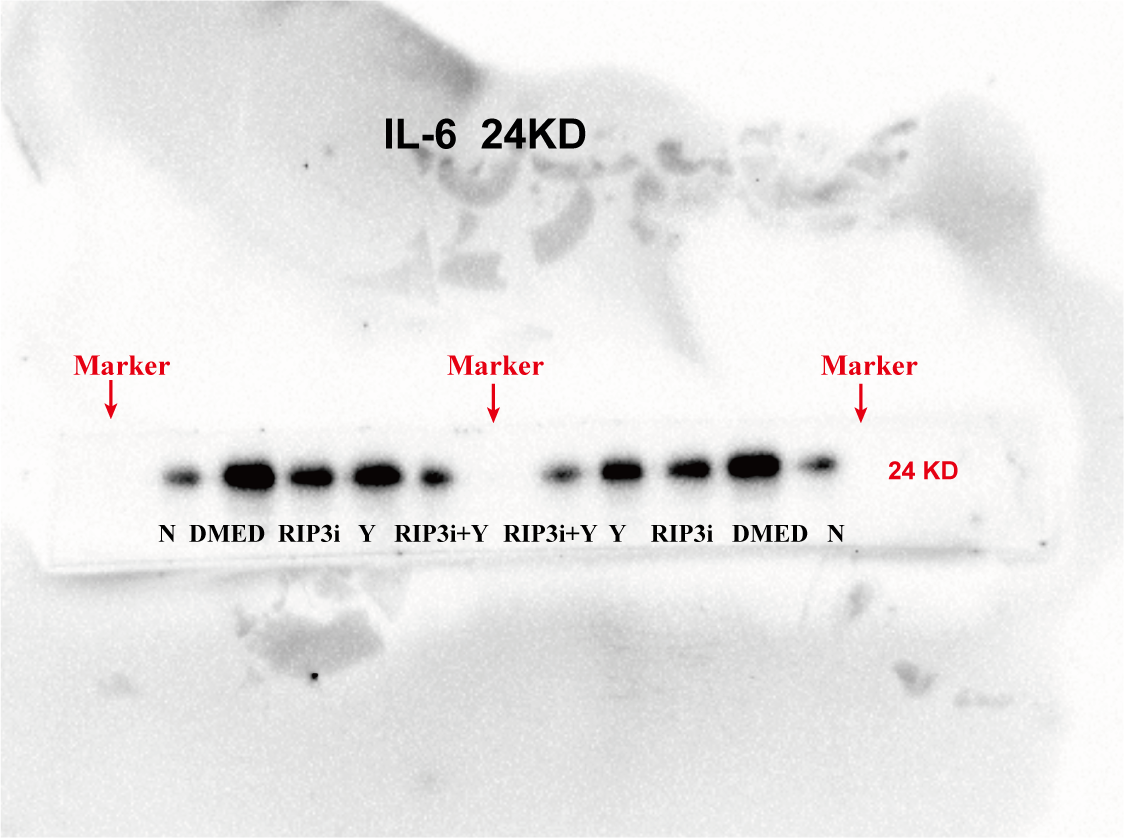

Supplement: Supplementary file 7 [file Image7.TIF]

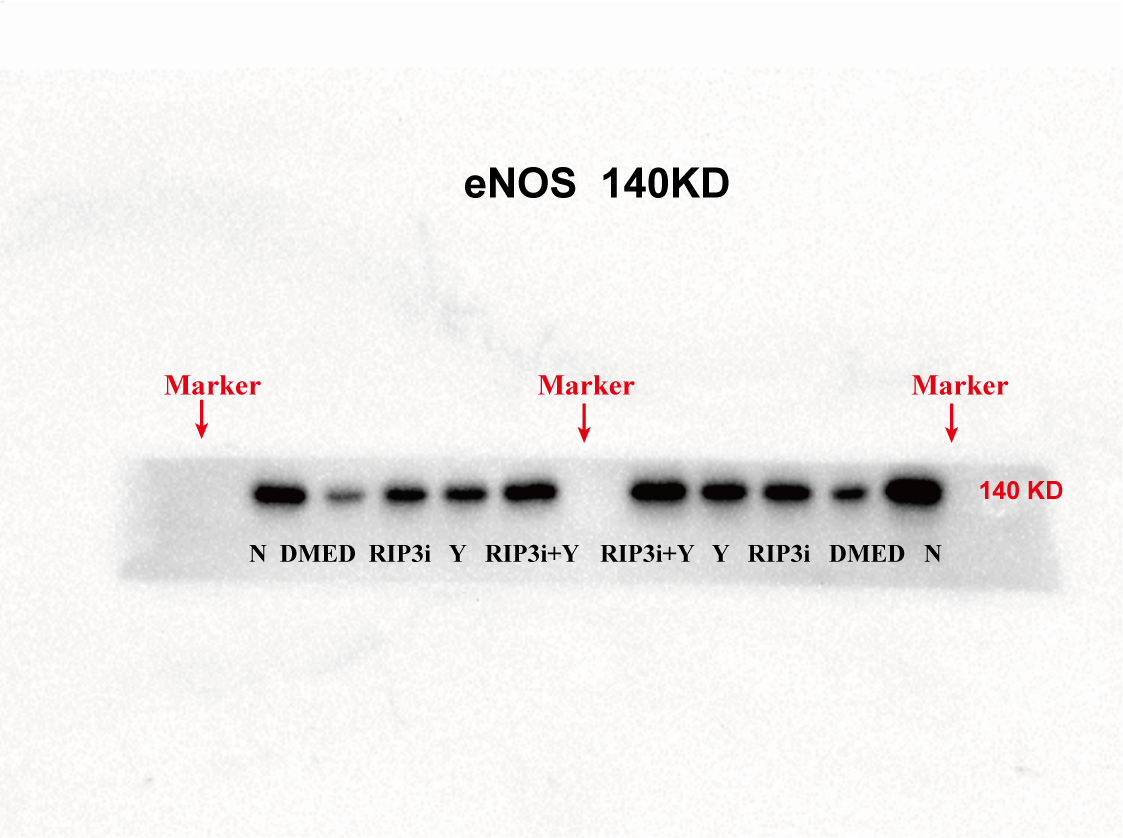

Supplement: Supplementary file 8 [file Image8.TIF]

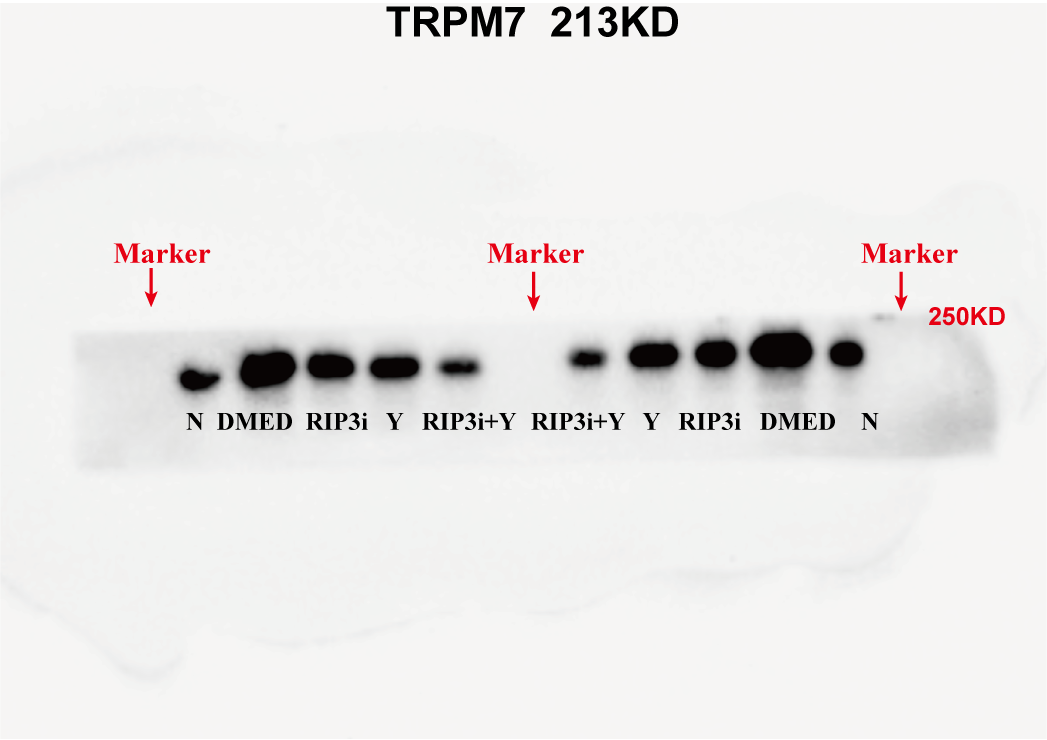

Supplement: Supplementary file 9 [file Image5.TIF]
